# Supplementary material for: Verification of radical pair mechanism predictions for weak magnetic field effects on superoxide in planarians
Source: bioRxiv. 2024 Nov 21:2024.11.20.624392. Preprint. [Version 1] doi: 10.1101/2024.11.20.624392 (PMC11601518; doi:10.1101/2024.11.20.624392)
Supplement: Supplement 1 [file NIHPP2024.11.20.624392v1-supplement-1.pdf]

bioRxiv preprint doi: https://doi.org/10.1101/2024.11.20.624392; this version posted November 21, 2024. The copyright holder for this preprint (which was not certified by peer review) is the author/funder, who has granted bioRxiv a license to display the preprint in perpetuity. It is made available under aCC-BY 4.0 International license.

Supporting Information

Multiple hyperfine interaction

We found that introducing a second HFI had significant effects, but adding additional HFIs beyond that had little impact.

Nuclei	HFCC (μT)
H5	-802.9
N5	431.3
H8 (X3)	255.4
N10	250.6
Hβ	190.8

Table 2: Hyperfine interactions for FH$^\bullet$ [30].

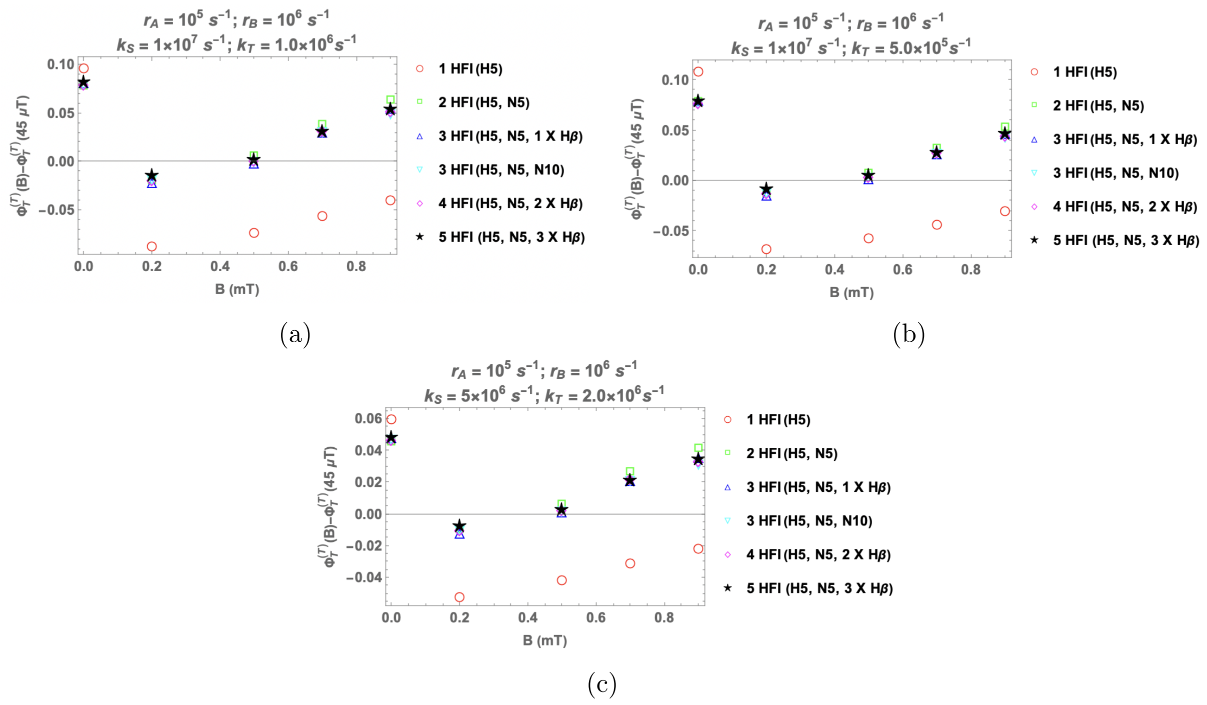

Figure 6: **Effects of having more than one HFIs:** Change in the fractional triplet yield for triplet-born RP with respect to the geomagnetic control (45 μT) as a function of the magnetic field. $r_A = 10^5\ s^{-1}$, $r_B = 10^6\ s^{-1}$. r_A and r_B are the spin relaxation rates of radicals A and B, respectively. k_S and k_T are singlet and triplet reaction rates, respectively.(a) $k_S = 10^7\ s^{-1}$ and $k_T = 10^6\ s^{-1}$, (b) $k_S = 10^7\ s^{-1}$ and $k_T = 5 \times 10^5\ s^{-1}$, (c) $k_S = 5 \times 10^6\ s^{-1}$ and $k_T = 2 \times 10^6\ s^{-1}$
